# Supplementary material for: The mouse cortico–basal ganglia–thalamic network
Source: Nature. 2021 Oct 6;598(7879):188–94. doi: 10.1038/s41586-021-03993-3 (PMC8494639; doi:10.1038/s41586-021-03993-3)
Supplement: Supplementary file 1 — This file contains Supplementary Tables 1 and 2. Supplementary Table 1: Striatal inputs to SNr domains by level. Supplementary Table 2: Striatal Inputs to GPe domains by level. [file 41586_2021_3993_MOESM1_ESM.pdf]

nature portfolio

https://doi.org/10.1038/s41586-021-03993-3

Supplementary information

The mouse cortico–basal ganglia–thalamic network

In the format provided by the authors and unedited

Table 1. Striatal inputs to SNr domains by level

SNr domains	*Striatal inputs to SNr domain*	*SNr levels*					
		ARA 81	*ARA 83*	*ARA 85*	*ARA 87*	*ARA 89*	*ARA 91*
anterolateral	CPi.vm.cvm	CPi.vm.cvm					
anteromedial	CPi.dm.cd	CPi.dm.cd					
central	CPi.dm.cd<br>CPi.vm.cvm		CPi.dm.cd<br>CPi.vm.cvm	CPi.dm.cd<br>CPi.vm.cvm			
centrolateral	CPi.vl.cvl<br>CPc.i.vm<br>CPc.i.vl		CPi.vl.cvl	CPi.vl.cvl<br>CPc.i.vm<br>CPc.i.vl			
dorsal	CPr.l.vm<br>CPi.vm.vm<br>CPi.vm.v<br>CPi.vm.cvm<br>CPc.v.vm<br>ACBc<br>ACBsh.l<br>ACBsh.m	CPr.l.vm<br>CPi.vm.vm<br>CPi.vm.v<br>ACBsh.l	CPr.l.vm<br>CPi.vm.vm<br>CPi.vm.v<br>ACBsh.l	CPi.vm.vm<br>CPi.vm.v<br>CPc.v.vm<br>ACBsh.l	CPi.vm.vm<br>CPi.vm.v<br>CPi.vm.cvm<br>ACBc<br>ACBsh.l<br>ACBsh.m	CPi.vm.vm<br>CPi.vm.v<br>CPi.vm.cvm<br>CPc.v.vm<br>ACBc<br>ACBsh.l	CPr.l.vm<br>CPi.vm.vm<br>CPi.vm.v<br>CPi.vm.cvm
dorsomedial	ACBc<br>ACBsh.m	ACBc<br>ACBsh.m	ACBc<br>ACBsh.m	ACBc<br>ACBsh.m			
external lateral	CPext.cv	CPext.cv			CPext.cv		
lateral	CPc.d.dm<br>CPc.d.vm<br>CPc.i.d<br>CPc.i.vl<br>CPc.i.vm<br>CPc.v.vl<br>CPc.v.vm<br>CPext.d<br>CPext.cv<br>CPext.rv	CPc.i.vl<br>CPc.i.vm<br>CPext.d<br>CPext.rv	CPc.d.vm<br>CPc.i.d<br>CPc.i.vm<br>CPext.d	CPc.i.d<br>CPext.d<br>CPext.cv	CPc.d.dm<br>CPc.d.vm<br>CPc.i.d<br>CPc.i.vl<br>CPc.i.vm<br>CPc.v.vl<br>CPext.d<br>CPext.rv	CPc.d.dm<br>CPc.d.vm<br>CPc.i.d<br>CPc.i.vm<br>CPext.d<br>CPext.cv	CPc.d.vm<br>CPc.v.vm<br>CPext.d<br>CPext.cv
lower limb	CPi.dl.imd (ll)<br>CPi.vl.cvl<br>CPc.d.dl<br>CPc.i.vl					CPi.dl.imd (ll)<br>CPi.vl.cvl<br>CPc.i.vl	CPi.dl.imd (ll)<br>CPc.d.dl
medial	CPr.m<br>CPr.imd<br>CPr.imv<br>CPr.l.ls<br>CPr.l.vm<br>CPi.dm.d<br>CPi.dm.dl<br>CPi.dm.dm<br>CPi.dm.im<br>CPi.dl.d (tr)<br>CPc.d.dm<br>ACBsh.l	CPr.m<br>CPr.imv<br>CPi.dm.d<br>CPi.dm.dm<br>CPi.dm.im<br>CPc.d.dm	CPr.m<br>CPr.imd<br>CPr.imv<br>CPr.l.ls<br>CPi.dm.d<br>CPi.dm.dl<br>CPi.dm.im	CPr.m<br>CPr.imd<br>CPr.imv<br>CPr.l.ls<br>CPr.l.vm<br>CPi.dm.d<br>CPi.dm.dl<br>CPi.dm.dm<br>CPi.dm.im	CPr.m<br>CPr.imd<br>CPr.imv<br>CPr.l.ls<br>CPi.dm.d<br>CPi.dm.dm<br>CPi.dm.im	CPr.m<br>CPr.imv<br>CPi.dm.d<br>CPi.dm.dm<br>CPi.dm.im	CPr.m<br>CPr.imd<br>CPr.l.ls<br>CPi.dm.d<br>CPi.dm.dl<br>CPi.dm.dm<br>CPi.dm.im<br>CPi.dl.d (tr)<br>CPc.d.dm<br>ACBsh.l
oro-brachial	CPr.l.vm<br>CPi.vl.imv (ul)<br>CPi.vl.v (m/i)<br>CPi.vl.vt (m/o)<br>CPi.vl.cvl<br>CPc.i.d<br>CPc.i.vl<br>CPc.v.vl<br>CPc.v.vm	CPi.vl.imv (ul)<br>CPc.v.vl	CPi.vl.imv (ul)<br>CPi.vl.v (m/i)<br>CPc.i.vl	CPi.vl.imv (ul)<br>CPi.vl.v (m/i)<br>CPi.vl.vt (m/o)	CPi.vl.imv (ul)<br>CPi.vl.v (m/i)<br>CPi.vl.vt (m/o)<br>CPc.v.vm	CPr.l.vm<br>CPi.vl.imv (ul)<br>CPi.vl.v (m/i)<br>CPi.vl.vt (m/o)	CPi.vl.imv (ul)<br>CPi.vl.v (m/i)<br>CPi.vl.vt (m/o)<br>CPi.vl.cvl<br>CPc.i.d<br>CPc.i.vl
orofacial	CPi.vl.vt (m/o)<br>CPc.v.vm	CPi.vl.vt (m/o)<br>CPc.v.vm	CPi.vl.vt (m/o)<br>CPc.v.vm				
paralateral	CPc.i.vm<br>CPc.v.vl<br>CPext.cv<br>CPext.rv		CPc.v.vl<br>CPext.cv<br>CPext.rv	CPc.v.vl<br>CPext.rv		CPc.v.vl<br>CPext.rv	CPc.i.vm<br>CPc.v.vl<br>CPext.rv
ventral	CPr.imd<br>CPr.l.ls<br>CPr.l.vm<br>CPi.dm.cd<br>CPi.dm.dl<br>CPi.dm.dm<br>CPi.dl.d (tr)<br>CPi.dl.imd (ll)<br>CPi.vl.cvl<br>CPc.d.dl<br>CPc.d.dm<br>CPc.d.vm<br>CPc.i.d	CPi.dm.dl<br>CPi.dl.d (tr)<br>CPi.vl.cvl<br>CPc.d.dl<br>CPc.d.vm<br>CPc.i.d	CPi.dm.dm<br>CPi.dl.d (tr)<br>CPc.d.dl<br>CPc.d.dm	CPi.dl.d (tr)<br>CPc.d.dl<br>CPc.d.dm<br>CPc.d.vm	CPr.imd<br>CPr.l.vm<br>CPi.dm.cd<br>CPi.dm.dl<br>CPi.dl.d (tr)<br>CPi.dl.imd (ll)<br>CPi.vl.cvl<br>CPc.d.dl	CPr.l.ls<br>CPi.dm.dl<br>CPi.dl.d (tr)<br>CPc.d.dl	

Supplementary Table 2. Striatal inputs to GPe domains by level

GPe domains	Striatal inputs to GPe domain	GPe levels ARA 58	ARA 60	ARA 62	ARA 64	ARA 66	ARA 68
1	CPi.vm.vm<br>CPc.i.vm<br>ACBc<br>ACBsh.m	CPi.vm.vm<br>ACBc<br>ACBsh.m	CPi.vm.vm<br>CPc.i.vm<br>ACBc				
2	CPr.imv	CPr.imv					
3	CPr.m<br>CPi.dm.d<br>CPi.dm.dm<br>CPi.dm.im<br>CPc.d.dm	CPr.m<br>CPi.dm.dm<br>CPi.dm.im	CPr.m<br>CPi.dm.d<br>CPi.dm.dm	CPi.dm.d<br>CPi.dm.dm<br>CPi.dm.im<br>CPc.d.dm			
4	CPr.imd<br>CPi.dm.d	CPr.imd<br>CPi.dm.d					
5	CPi.dm.cd<br>CPi.vm.cvm<br>CPc.d.dm	CPi.dm.cd<br>CPi.vm.cvm<br>CPc.d.dm	CPi.vm.cvm<br>CPc.d.dm				
6	CPr.imd<br>CPi.dl.d (tr)<br>CPi.dl.d.r<br>CPi.dm.dl<br>CPi.vl.cvl<br>CPc.d.dl	CPi.dl.d.r<br>CPi.dm.dl<br>CPi.vl.cvl	CPr.imd<br>CPi.dl.d (tr)<br>CPi.dl.d.r<br>CPi.dm.dl<br>CPc.d.dl				
7	CPr.l.ls	CPr.l.ls					
8	CPi.vl.imv.r<br>CPc.i.vm	CPi.vl.imv.r<br>CPc.i.vm					
9	CPi.vl.v.r<br>CPi.vl.vt (m/o)	CPi.vl.v.r<br>CPi.vl.vt (m/o)	CPi.vl.vt (m/o)	CPi.vl.vt (m/o)			
10	CPr.l.vm<br>CPi.vm.v<br>CPc.v.vm<br>ACBsh.l<br>ACBsh.m	CPr.l.vm<br>CPi.vm.v<br>ACBsh.l	CPi.vm.v<br>CPc.v.vm<br>ACBsh.l<br>ACBsh.m				
11	CPc.v.vm<br>CPext.cv	CPc.v.vm		CPc.v.vm	CPc.v.vm	CPc.v.vm<br>CPext.cv	
12	CPr.imv<br>CPi.dm.cd<br>CPi.dm.im		CPr.imv<br>CPi.dm.cd<br>CPi.dm.im				
13	CPr.l.ls<br>CPr.l.vm		CPr.l.ls<br>CPr.l.vm				
14	CPi.vl.cvl<br>CPc.d.vm		CPi.vl.cvl<br>CPc.d.vm				
15	CPi.dl.imd (ll)<br>CPi.vl.imv.r		CPi.dl.imd (ll)<br>CPi.vl.imv.r				
16	CPi.vl.imv (ul)		CPi.vl.imv (ul)	CPi.vl.imv (ul)	CPi.vl.imv (ul)		
17	CPi.vl.v (m/i)<br>CPi.vl.v.r		CPi.vl.v (m/i)<br>CPi.vl.v.r	CPi.vl.v (m/i)<br>CPi.vl.v.r	CPi.vl.v (m/i)		
18	CPr.imd<br>CPr.l.ls<br>CPi.vm.v<br>CPi.vm.vm			CPr.imd<br>CPr.l.ls<br>CPi.vm.v<br>CPi.vm.vm			
19	ACBsh.l			ACBsh.l			
20	CPr.imv<br>CPi.vm.cvm<br>CPc.i.vm			CPr.imv<br>CPi.vm.cvm<br>CPc.i.vm			
21	CPi.dm.cd			CPi.dm.cd			
22	CPc.d.vm			CPc.d.vm			
23	CPi.dl.d.r<br>CPi.dm.dl			CPi.dl.d.r<br>CPi.dm.dl			
24	CPi.vl.cvl<br>CPc.d.dl			CPi.vl.cvl<br>CPc.d.dl			
25	CPi.dl.d (tr)<br>CPi.dl.imd (ll)<br>CPi.vl.imv.r<br>CPc.i.d<br>CPc.i.vl			CPi.dl.d (tr)<br>CPi.dl.imd (ll)<br>CPi.vl.imv.r<br>CPc.i.d<br>CPc.i.vl			
26	CPi.dm.dm<br>CPc.d.dl<br>CPc.d.dm<br>CPc.i.d				CPi.dm.dm<br>CPc.d.dl<br>CPc.d.dm<br>CPc.i.d		
27	CPi.dl.d (tr)<br>CPi.dm.dl<br>CPi.vl.cvl<br>CPext.d				CPi.dl.d (tr)<br>CPi.dm.dl<br>CPi.vl.cvl<br>CPext.d		
28	CPi.dl.imd (ll)				CPi.dl.imd (ll)		
29	CPc.i.vl				CPc.i.vl		
30	CPr.l.vm<br>CPi.vl.vt (m/o)<br>CPc.v.vl<br>CPext.rv				CPr.l.vm<br>CPi.vl.vt (m/o)<br>CPc.v.vl<br>CPext.rv	CPr.l.vm<br>CPc.v.vl<br>CPext.rv	
31	CPc.d.dm					CPc.d.dm	
32	CPc.d.dl<br>CPc.d.vm<br>CPc.i.d<br>CPc.i.vm<br>CPext.d					CPc.d.dl<br>CPc.d.vm<br>CPc.i.d<br>CPc.i.vm<br>CPext.d	
33	CPc.v.vm<br>CPext.cv						CPc.v.vm<br>CPext.cv
34	CPc.d.dm<br>CPc.d.vm<br>CPc.i.vm						CPc.d.dm<br>CPc.d.vm<br>CPc.i.vm
35	CPext.d						CPext.d
36	CPext.rv						CPext.rv
