# Supplementary material for: The mouse cortico–basal ganglia–thalamic network
Source: Nature. 2021 Oct 6;598(7879):188–94. doi: 10.1038/s41586-021-03993-3 (PMC8494639; doi:10.1038/s41586-021-03993-3)
Supplement: Supplementary file 2 — Reporting Summary [file 41586_2021_3993_MOESM2_ESM.pdf]

nature research

Corresponding author(s): Hong-Wei Dong
Nicholas N. Foster

Last updated by author(s): Aug 26, 2021

Reporting Summary

Nature Research wishes to improve the reproducibility of the work that we publish. This form provides structure for consistency and transparency in reporting. For further information on Nature Research policies, see our Editorial Policies and the Editorial Policy Checklist.

Statistics

For all statistical analyses, confirm that the following items are present in the figure legend, table legend, main text, or Methods section.

n/a	Confirmed	
☐	☒	The exact sample size (n) for each experimental group/condition, given as a discrete number and unit of measurement
☐	☒	A statement on whether measurements were taken from distinct samples or whether the same sample was measured repeatedly
☐	☒	The statistical test(s) used AND whether they are one- or two-sided<br>*Only common tests should be described solely by name; describe more complex techniques in the Methods section.*
☒	☐	A description of all covariates tested
☐	☒	A description of any assumptions or corrections, such as tests of normality and adjustment for multiple comparisons
☐	☒	A full description of the statistical parameters including central tendency (e.g. means) or other basic estimates (e.g. regression coefficient) AND variation (e.g. standard deviation) or associated estimates of uncertainty (e.g. confidence intervals)
☐	☒	For null hypothesis testing, the test statistic (e.g. F, t, r) with confidence intervals, effect sizes, degrees of freedom and P value noted<br>*Give P values as exact values whenever suitable.*
☒	☐	For Bayesian analysis, information on the choice of priors and Markov chain Monte Carlo settings
☒	☐	For hierarchical and complex designs, identification of the appropriate level for tests and full reporting of outcomes
☒	☐	Estimates of effect sizes (e.g. Cohen's d, Pearson's r), indicating how they were calculated

Our web collection on statistics for biologists contains articles on many of the points above.

Software and code

Policy information about availability of computer code

Data collection	All epifluorescence images used in the striatal output axonal analyses were collected with the Olympus VS120 fluorescence microscope running Olympus VS-Desktop v2.9. High resolution confocal images were captured using an Andor DragonFly 202 spinning disk confocal microscope running Fusion v2.1.0.81 software. Lightsheet images were captured with a LifeCanvas lightsheet microscope running SmartSPIM Acquisition Software 2019v3. Electrophysiological data were collected using a MultiClamp700B Amplifier (Molecular Devices) running pClamp v. 10.7.
Data analysis	All standard statistical analyses were performed with GraphPad Prism v4.0c for Macintosh, including ANOVA, 2-sided t test with Welch's correction, Fisher's exact test, Pearson's r, and descriptive statistics. The algorithm implementing the Louvain analysis was obtained from the Brain Connectivity Toolbox (available at: https://sites.google.com/site/bctnet/) and executed in Python v2.7.<br><br>The Connection Lens v2.5.1 software used to register, threshold, and quantify the striatofugal axonal data was designed in-house. This software has not been released publicly yet, although it has been used in our previously published works (Hintiryan et al. 2016; Benavidez et al. 2021; Hintiryan et al. 2021).<br><br>Neurons from 3D images were reconstructed with Aivia v8.8.2, post-processed with the Quantitative Imaging Toolkit (available at: http://cabeen.io/qitwiki), and analyzed with neuTube v1.0z.<br><br>Electrophysiological signals were analyzed using Clampfit v. 10.7.

For manuscripts utilizing custom algorithms or software that are central to the research but not yet described in published literature, software must be made available to editors and reviewers. We strongly encourage code deposition in a community repository (e.g. GitHub). See the Nature Research guidelines for submitting code & software for further information.

Data

Policy information about availability of data

All manuscripts must include a data availability statement. This statement should provide the following information, where applicable:

- Accession codes, unique identifiers, or web links for publicly available datasets
- A list of figures that have associated raw data
- A description of any restrictions on data availability

Downsampled images of all data used in the striatofugal analyses are shown in Extended Data Figure 2. The quantified data for these cases can be accessed through our B.R.A.I.N. Lab website (http://brain.neurobio.ucla.edu/publications/). Also available are the SNr neuronal reconstructions from Figure 1k, the Supplementary Video, and an application presenting the projection maps of all axonal reconstructions.

Field-specific reporting

Please select the one below that is the best fit for your research. If you are not sure, read the appropriate sections before making your selection.

☒ Life sciences ☐ Behavioural & social sciences ☐ Ecological, evolutionary & environmental sciences

For a reference copy of the document with all sections, see nature.com/documents/nr-reporting-summary-flat.pdf

Life sciences study design

All studies must disclose on these points even when the disclosure is negative.

Sample size

The sample size for the striatofugal analysis was determined by the number of domains in the striatum, i.e., 36. This consists of 29 domains in the dorsal striatum as described in Hintiryan et al (2016), as well as 4 additional dorsal striatal domains described in Methods and 3 domains in the ventral striatum: the core, medial shell, and lateral shell. The ventral striatum may or may not contain more subregions, but the core and shell are well-documented sub-compartments of the accumbens, and differences in connectivity patterns of medial and lateral shell have been described (e.g., Wright, Beijer & Groenewegen 1996, J Neurosci). Data were accumulated until each domain was injected with a distinct, isolated tracer deposit. All together, 138 animals received 1-3 anterograde tracer injections in the striatum. The grand total sample size of 268 reported in the Methods section includes these 138 plus animals with injections with injections targeting other parts of the cortico-basal ganglia-thalamic network.

Sample sizes for the electrophysiology experiments are comparable to previously published works both for number of subjects used and for number of neurons recorded per group (Thorn, Atallah et al. 2010, Neuron; Ji, Zingg et al. 2016, Cerebral Cortex).

Data exclusions

One and only one injection per striatal domain was used in the striatofugal analysis, as is standard in neuroanatomical research and as is necessary for the kind of community analysis we conducted. The best, most representative injection for each domain was chosen for the analysis. The others were excluded due to off-targeting of the injection site, missing/damaged tissue in the pallidal and nigral regions of interest, and weak tracer labeling of the axons or high background.

For electrophysiology experiments, data were excluded from neurons that were recorded outside of the target nuclei (i.e., the GPe, SNr, MOp-m/i, and ACA).

Replication

All striatal domains were targeted with injections multiple times to generate the striatofugal dataset. While the best, most representative cases were chosen for inclusion in the analysis data set, the other injections served as validation cases, demonstrating the replicability and consistency of labeling arising from each domain.

Randomization

Randomization is not relevant to the present work since animals were not compared across different conditions.

Blinding

Traditional blinding was not necessary since animals were not compared across different conditions. However, bias in image registration for the striatofugal analysis is the one area where the methods could have affected the results. In that regard, the image registration process, although not technically blinded, was performed by contributors without any a priori knowledge of the pathways under investigation.

Reporting for specific materials, systems and methods

We require information from authors about some types of materials, experimental systems and methods used in many studies. Here, indicate whether each material, system or method listed is relevant to your study. If you are not sure if a list item applies to your research, read the appropriate section before selecting a response.

Materials & experimental systems

n/a	Involved in the study	
☐	☒	Antibodies
☒	☐	Eukaryotic cell lines
☒	☐	Palaeontology and archaeology
☐	☒	Animals and other organisms
☒	☐	Human research participants
☒	☐	Clinical data
☒	☐	Dual use research of concern

Methods

n/a	Involved in the study	
☒	☐	ChIP-seq
☒	☐	Flow cytometry
☒	☐	MRI-based neuroimaging

Antibodies

Antibodies used	[antibody; vendor; catalog number]<br>1. rabbit anti-Phaseolus vulgaris leucoagglutinin antibody; Vector Labs; #AS-2300<br>2. monoclonal mouse anti-Cre recombinase, clone 2D8; Millipore Sigma; #MAB3120<br>3. donkey anti-rabbit AlexaFluor647 antibody; Jackson ImmunoResearch, #711-605-152<br>4. donkey anti-mouse AlexaFluor647 antibody; Jackson ImmunoResearch, #715-605-150
Validation	Supporting documentation as to the validity of the above antibodies can be found at the following:<br>1. https://antibodyregistry.org/search.php?q=AB_2313686 ; see also Gerfen & Sawchenko, 2016, Brain Research<br>2. https://antibodyregistry.org/search.php?q=AB_2085748 and https://www.emdmillipore.com/US/en/product/Anti-Cre-Recombinase-Antibody-clone-2D8,MM_NF-MAB3120#documentation ; we have also used it previously in Benavidez et al. 2021 and Hintiryan et al. 2021

Animals and other organisms

Policy information about studies involving animals; ARRIVE guidelines recommended for reporting animal research

Laboratory animals	Mus musculus, male, 2-month old, wild type C57Bl6 and Ai14 (007908), obtained from Jackson Laboratories
Wild animals	No wild animals were used in this study.
Field-collected samples	No field samples were collected for this study.
Ethics oversight	Ethical oversight of experimental procedures was performed by the Institutional Animal Care and Use Committee (IACUC) of the University of California, Los Angeles, IACUC at the University of Southern California, IACUC at the University of California, San Diego, and the Institutional Ethics Committee of Huazhong University of Science and Technology.

Note that full information on the approval of the study protocol must also be provided in the manuscript.
